# Surgical Club of South West England

**Published:** 1988-02

**Authors:** 


### Surgical Club of South West England

### Meeting in Bath, October 1986

### THE MANAGEMENT OF DIABETIC GANGRENE

A. R. Turnbull
Royal United Hospital, Bath

All partial amputations of the foot for diabetic gangrene were reviewed from April 1981 to March 1986. There were 68 patients with a mean age of 69 years. 40 (59%) were male, 28 (41%) were female; 37 (54%) were insulin-dependent, and 31 (46%) were non-insulin-dependent. There were 103 primary operations which included 6 debridements, 35 raquet, 52 ray, 4 transmetatarsal, and 6 heel amputations. 68/103 (66%) healed with no further surgery. 5/68 (7%) patients underwent 7 femoro-popliteal bypass operations, 18/68 (26%) healed after further surgery, 12/68 (18%) underwent leg amputation, and 5/68 (7%) died. Analysis of the patients requiring re-operation showed that 90% of the original operations had been performed by a surgeon in training. Healing took from 1–30 months and ray amputations took longer (mean 7 months) than raquet amputations (mean 4 months). Primary healing was more frequent when foot pulses were present and no patient with foot pulses came to leg amputation. There was no difference in outcome between insulin-dependent and non-insulin-dependent diabetics. Overall limb salvage was achieved in 79% of cases.

### FINE NEEDLE ASPIRATION CYTOLOGY OF BREAST MASSES

Paul Gartell
Royal United Hospital, Bath

Aspiration cytology of breast masses has many advantages that favour its use. However, its major disadvantage is a high rate of aspirates which are acellular or heavily blood stained and hence unsatisfactory for diagnostic purposes.

In a retrospective study of 1663 breast aspirations in 1458 patients in Southampton, the overall proportion of unsatisfactory aspirates from breast masses was 42%. Invasive lobular carcinomas yielded a significantly higher rate of unsatisfactory aspirates (63%) than invasive ductal carcinoma (26%) ($P<.001$) and similarly fibroadenoma yielded a significantly lower unsatisfactory aspiration rate (21%) than fibroadenosis (56%) ($P<0.001$). The proportion of unsatisfactory aspirates was inversely related to tumour size in patients with invasive ductal carcinoma or fibroadenoma, but not for invasive lobular carcinoma or fibroadenosis.

In a prospective trial of 96 patients to assess the value of ultrasound localisation for aspiration cytology of breast lumps, the proportion of unsatisfactory aspirates from breast masses having ultrasound-guided aspiration cytology (22%) was significantly lower than the unsatisfactory rate of aspiration cytology in clinic (41%). The difference was most marked in patients with a mass less than 3 cms. in diameter. The results were influenced by the experience of the clinician performing the aspiration and the number of needle manoeuvres performed.

We conclude that although experience and technique are the most important factors in obtaining a satisfactory aspirate, consideration should be given to using ultrasound localisation in patients with a tumour less than 3 cms. in diameter.

### URINARY FIBRINOLYSIS AND STONE FORMATION

Clive Charlton
Royal United Hospital, Bath

It seems likely, that within the urinary tract there exists a mechanism which is in part responsible for maintaining its patency, and that this is achieved by degrading debris and detritus into smaller aggregates, so facilitating their elimination from the tubular system. The dissolution of blood clot by the action of the urinary fibrinolytic system is an example of this phenomenon.

In normal urine, about 0.5 gram of large molecular weight substances (thereafter referred to as colloids) are excreted per day, the largest (Tamm-Horsfall muco-proteins) measuring 28 million daltons, and the smaller ones less than one thousand daltons. I have previously demonstrated that the proteolytic fibrinolytic system is effective in degrading these colloids, and so the larger colloids are broken down into smaller aggregates which consequently result in an increase in their surface area. In the normal individual, two-thirds of the urinary macro-molecules are smaller than 30,000 daltons, yet in stone formers urine, all macromolecules are larger than 100,000 daltons, and so the colloidal surface area available for the chelation or absorption of calcium is diminished (the colloids are negatively charged particles) as compared with an equal amount of the colloid in normal urine.

The urinary fibrinolytic activity of 188 stone formers urine as compared to an equal number of matched controls is significantly reduced, and could therefore be the reason for the presence of the larger colloid found in the stone formers urine.

I am suggesting that it is due to decreased urinary fibrinolytic activity that the urinary colloids of stone formers urine are larger than those found in an equal amount of colloid in the urine of normal individuals; and since this results in a decrease in the total surface area of these colloids, it follows that a lesser amount of calcium can be absorbed on to the colloidal surface and this permits super saturation and so precipitation of crystalloids results. Hence, although the amount of calcium in the urines of the stone formers and controls may be the same, in the former situation concrement formation is favoured, and this disorder can be avoided by restoring the urinary fibrinolytic activity to its normal physiological levels.

### GALL STONES

W F W Southwood
Royal United Hospital, Bath

The increasingly common problem of gall stones was discussed with particular reference to the patients presenting themselves in Bath with this condition. These are currently around 30 per month.

The first hundred patients of 1986 were reviewed. In most instances confirmatory tests had been performed by the referring practitioner 53 had ultrasound, 40 cholecystography and 2 a hidascan.

The operations performed were described and particular emphasis was laid on the importance of operative cholangiography which was performed in 72%. Although the standard technique using films can be employed the procedure can be speeded if cinefluoroscopy and an image intensifier are used. This gives a reliable picture and if dye fails to enter the duodenum, it can usually be made to do so by injecting more than the customary 10 ml. Antispasmodic drugs are often therefore not required.

Exploration of the bile duct was performed in 18 patients and stones were retrieved from 16 of these. 9 of these patients underwent choledochoscopy and there were 2 incidents of retained calculi, one of whom had choledochoscopy.

### REGISTRAR'S PRIZE PAPERS

*The following three papers were submitted for the Registrar's Prize.*

### THE LATERAL THYROID LIGAMENT

**C. K. Leow, M. H. Thompson**
**Southmead Hospital, Bristol**

The recurrent laryngeal nerve (RLN) lies in close contact with the lateral ligament of the thyroid gland. Division of the ligament is essential for complete thyroid lobectomy. Accidental RLN injury following thyroidectomy varies from 0.3% to more than 13% (Loré et al.). Surprisingly, the majority of British and American anatomical and surgical literature has no mention of the extent and relationship of this ligament to the RLN.

Detailed dissection of the thyroid gland and the adjacent region was carried out on ten cadavers and fifteen post-mortem specimens.

All twenty-five cases possessed the lateral thyroid ligament which was attached to the postero-medial glandular surface of the superior thyroid lobe. Posteriorly, the ligament was attached to the inferior margin of the cornu of the cricoid cartilage, near its pole; it then extended infero-medially onto the antero-lateral tracheal wall. In all cases the RLN was found to lie on the antero-lateral surface of this ligament and not embedded within it.

To preserve the RLN, Loré recommended the transection of the ligament as close to the trachea and cricoid cartilage as possible. The proximity of the RLN to the ligament at the cricoid cartilage level as described in this study suggest it may be safer to transect the ligament close to the gland.

### THE IN VIVO STAGING OF RECTAL CANCER WITH RECTAL SONOGRAPHY

**J. Beynon, N. J. McMortensen**
**Bristol Royal Infirmary**

Five ultrasonic layers have been identified in the rectum; a first echogenic layer which was mucosa, an echopoor layer representing mucosa and muscularis mucosae, an echogenic layer which was submucosa, echopoor layer which was muscularis propria and an echogenic layer which was pararectal fat or serosa.

Endoluminal ultrasound's (ELU) effectiveness in the staging of rectal cancer has been assessed in 82 patients who were also graded digitally and in some cases (44) by computed tomography (CT).

Digital examination had an accuracy of 57%. ELU staging of local invasion when compared to histopathology had a correlation coefficient (r) of 0.88 ($p<0.001$). ELU's accuracy was 92% and it predicted invasion beyond the muscularis propria with a sensitivity of 97%, specificity of 94%, positive predictive value (PPV) of 98% and negative predictive value (NPV) of 89%. In the comparative study ELU was the most accurate, CT having an accuracy of 82%, sensitivity of 86%, specificity of 62%, PPV of 91% and NPV of 50%. In 35 patients ELU tumour depth was compared with maximum depth on histological section, $r=0.63$ ($p<0.001$). In 27 patients ELU was compared with depths measured from the resected specimen, $r=0.80$ ($p<0.001$).

Pre-operative assessments of rectal tumours with ELU accurately stages local invasion and provides an objective method of differentiating between inflammatory and malignant infiltration.

### FINE-NEEDLE ASPIRATION BIOPSY FOR THE DIAGNOSIS OF THYROID CANCER

**J. B. Anderson, A. J. Webb, (R. C. N. Williamson)**
**Bristol Royal Infirmary, Bristol**

Conventional criteria for the evaluation of isolated thyroid swellings are inaccurate in identifying the small proportion of malignant tumours amongst the many benign lesions presenting in clinical practice. The diagnostic accuracy of fine-needle aspiration biopsy (FNAB) for cytology was therefore assessed in 562 patients with nodular thyroid disease, 373 of whom (66.4%) had histopathological confirmation of the cytological diagnosis.

Sixty-one aspiration biopsies were positive for malignancy, and the diagnosis was confirmed histologically in 59 of these (96.7%). Thus there were 2 false positive cytology results among 310 patients with proven benign disease (0.7%). Four of 63 patients with proven carcinoma had a benign cytological diagnosis, a false negative rate of 6.3%. In 57 of the 59 malignancies correctly diagnosed by FNAB (97%) the histological type of tumour was successfully identified. Overall 367 of 373 patients received correct cytological discrimination between benign and malignant nodules, an overall accuracy for FNAB of 98.4%. The sensitivity of the test for the detection of malignancy was 93.7% and the specificity for benign conditions 99.3%.

Although FNAB is safe, cost-effective and reliable in establishing the malignant nature and type of thyroid carcinoma, its use has remained limited in this county for ill-defined reasons (1). Its superior diagnostic reliability allows the appropriate selection of patients for surgery and ensures the correct operation is performed for each type of tumour.

#### REFERENCE

1. Fox C. H. Innovation in medical diagnosis—the Scandinavian curiosity. Lancet 1979; i: 1398–8.

